# The Mitochondrial Routing of the Kv1.3 Channel

**DOI:** 10.3389/fonc.2022.865686

**Published:** 2022-03-24

**Authors:** Jesusa Capera, María Navarro-Pérez, Anne Stine Moen, Ildiko Szabó, Antonio Felipe

**Affiliations:** ^1^ Molecular Physiology Laboratory, Dpt. de Bioquímica i Biomedicina Molecular, Institut de Biomedicina (IBUB), Universitat de Barcelona, Barcelona, Spain; ^2^ Kennedy Institute of Rheumatology, University of Oxford, Oxford, United Kingdom; ^3^ Department of Biology, University of Padova, Padova, Italy

**Keywords:** potassium channels, mitochondria, apoptosis, TIM-TOM complex, cancer

## Abstract

Voltage-gated potassium channels control neuronal excitability and cardiac action potentials. In addition, these proteins are involved in a myriad of cellular processes. The potassium channel Kv1.3 plays an essential role in the immune response mediated by leukocytes. Kv1.3 is functional both at the plasma membrane and the inner mitochondrial membrane. Plasma membrane Kv1.3 mediates cellular activation and proliferation, whereas mitochondrial Kv1.3 participates in cell survival and apoptosis. Therefore, this protein emerges as an important target in cancer therapies. Several forward-traffic motifs target the channel to the plasma membrane in a COPII-dependent manner. However, the mitochondrial import pathway for Kv1.3 is largely unknown. Here, we deciphered the mitochondrial routing of the mitoKv1.3 channel. Kv1.3 uses the TIM23 complex to translocate to the inner mitochondrial membrane. This mechanism is unconventional because the channel is a multimembrane spanning protein without a defined N-terminal presequence. We found that transmembrane domains cooperatively mediate Kv1.3 mitochondrial targeting and identified the cytosolic HSP70/HSP90 chaperone complex as a key regulator of the process. Our results provide insights into the mechanisms mediating the localization of Kv1.3 to mitochondrial membranes, further extending the knowledge of ion channel biogenesis and turnover in mitochondria.

## Introduction

Mitochondria fuel cellular metabolism. Their role in energy production culminates with oxidative phosphorylation. The mitochondrial membrane potential (ΔΨm) is highly negative on the matrix side (150-180 mV), and along with the proton gradient, it generates the driving force for ATP synthesis. Maintenance of the ΔΨm depends on the compartmentalization of the inner mitochondrial membrane (IMM) in domains forming cristae. Mitochondria are highly dynamic organelles that undergo fusion or fission in a tightly regulated manner to modulate their function. The outer mitochondrial membrane (OMM) contacts other organelles, such as the endoplasmic reticulum (ER) or the plasma membrane (PM), and forms intermitochondrial junctions. Therefore, mitochondria have an increasing number of functions beyond energy homeostasis. These processes include apoptosis, cell cycle progression and autophagy, among others (1, 2). Mitochondria depend on the import of nuclear DNA-encoded proteins to perform their functions, since only a few mitochondrial proteins are encoded in the mitochondrial genome (3).

Different import pathways exist that depend on the intramitochondrial destination of the protein (OMM, IMM, intermembrane space (IMS) and matrix). Briefly, nuclear DNA-encoded proteins are synthesized on cytosolic ribosomes and approach the OMM either co or posttranslationally (4). Cytosolic chaperone complexes participate in delivering the protein to the receptors of the translocase of the outer membrane (TOM) in a partially folded import-competent state (5). Target proteins are transferred to the OMM or the IMS through different mechanisms involving the sorting and assembly machinery (SAM) and the mitochondrial IMS import and assembly machinery (MIA), respectively. Alternatively, proteins are further translocated across the IMM through the translocases of the inner membrane (TIM23 and TIM22). Matrix-resident proteins are imported with the help of a presequence translocase-associated motor (PAM) after TIM translocation (6, 7). Most mitochondrial proteins contain a targeting presequence at the N-terminal domain, which is an amphipathic α-helix that is cleaved once the protein arrives at the destination. In contrast, for many multimembrane spanning proteins of the IMM, the targeting presequence is absent. In some cases, these proteins contain internal signals that are widely distributed in the protein sequence. How these domains cooperate to drive the protein to the IMM and contribute to the proper folding and membrane topology of the protein remains obscure. The TIM23 complex mediates the import of proteins with the presequence, while the TIM22 complex is responsible for N-terminal presequence-independent protein import (8). Interestingly, some mitochondrial proteins also target other destinations within the cell (9). Thus, mechanisms of mitochondrial routing are relevant and may determine protein function. One example of this phenomenon is mitochondrial ion channels.

Several ion channels target both the PM and mitochondria (10, 11). Their role in mitochondria involves the regulation of the ΔΨm, production of reactive oxygen species (ROS) and regulation of Ca^2+^ signaling (12, 13). Although the mechanisms mediating the translocation of these proteins into the mitochondrial membranes are generally unknown, mitochondrial targeting sequences can be added through alternative splicing. For instance, a short isoform of Kir1.1 contains a canonical N-terminal presequence that targets the protein to the IMM, forming mitoKATP channels (14–16). Furthermore, mitoBK_Ca_ channels translocate to the IMM through alternative splicing at the C-terminus of KCa1.1. However, alternative splicing mechanisms are not unique because uniexonic voltage-gated potassium channels of the *Shaker* family, such as Kv1.1, Kv1.3 and Kv1.5, target the IMM (17–19). On the other hand, the mitochondrial sorting of Kesv, a viral K^+^ channel with only two transmembrane segments, depends on unidentified signals within the C-terminal transmembrane hydrophobic domain that are recognized by the TIM/TOM complex (20).

In this scenario, the essential role of mitoKv1.3 has been extensively studied, but the mechanisms of its mitochondrial targeting remain elusive. MitoKv1.3 is linked to the control of apoptosis through a Bax-dependent mechanism, with important repercussions for anticancer therapy (21). In addition, mitoKv1.3 controls cell cycle progression by regulating mitochondrial dynamics (18). Factors determining the equilibrium between PM and mitochondrial Kv1.3 are scarcely known (19), although knowledge of these factors would be essential to understand the pathophysiology of many processes involved in human Kv1.3-related illnesses, such as cancer, autoimmune diseases and obesity (22).

In the present study, we elucidated the mitochondrial import pathway of Kv1.3. HSP70/HSP90 cytosolic chaperones route the channel to the OMM, where it translocates *via* an unconventional TIM/TOM mechanism. Despite the lack of an evident mitochondrial targeting sequence, Kv1.3 is imported through the TIM23 translocase complex of the IMM. We also deciphered that the transmembrane domains of Kv1.3 function as redundant and complementary mediators of mitochondrial targeting. Furthermore, we suggest that members of the *Shaker* family of voltage-gated potassium channels share this mechanism. Our work provides insights into the mitochondrial import mechanisms for large and multispanning membrane proteins lacking evident mitochondrial targeting sequences and provides insights into the control of the PM/mitochondrial equilibrium of voltage-gated potassium channels.

## Materials and Methods

### Expression Plasmids, Constructs and Site-Directed Mutagenesis

Rat Kv1.3 and human Kv1.5 channels and the mitochondrial marker have been widely described by our laboratory (18, 19, 23, 24). Rat Kv1.1, Kv1.2 and Kv1.4 channels in pGEM7 were obtained from M. M. Tamkun (Colorado State University, CO). Channel cDNAs were subcloned into pEYFP-C1 (Clontech). HA-Sar1 (H79G) was from R. Pepperkok (EMBL, Heidelberg, Germany). Kv1.3-ΔSx constructs were generated by inserting XhoI sites at the beginning and at the end of the Sx sequence in the Kv1.3 pEYFP-C1 plasmid. Mutations to introduce restriction sites were generated using the QuikChange multisite-directed mutagenesis kit (Agilent Technologies). YFP-Sx constructs were obtained by fusing the Sx segment to the C-terminus of pEYFP-C1 (Clontech). Forward and reverse oligonucleotide strands containing the Sx sequence were designed with EcoRI and BamHI sites at the beginning and at the end, respectively, to generate Sx segments. Oligonucleotides were annealed and cloned into the pEYFP-C1 plasmid. Primers designed to obtain the constructs are shown in **Supplementary Tables 1** and **2**. All constructs were verified by sequencing.

### Cell Culture, Transient Transfection and Drug Treatments

HEK 293 cells were grown in DMEM containing 10% FBS and 100 U/ml penicillin/streptomycin (Gibco). Transient transfection was performed using LipoTransfectin (Attendbio) in cells after reaching approximately 80% confluence, and experiments were performed 24 h after transfection. All drugs were dissolved in dimethyl sulfoxide (DMSO) and diluted in DMEM to a final DMSO concentration of <0.5%. Ver-155008 and 17-DMAG were purchased from Sigma.

### Protein Extraction, Coimmunoprecipitation and Western Blot Analysis

Cells were washed twice with cold PBS and lysed on ice with lysis solution (1% Triton X-100, 10% glycerol, 50 mM HEPES, and 150 mM NaCl, pH 7.2) supplemented with 1 mg/ml aprotinin, 1 mg/ml leupeptin, 1 mg/ml pepstatin, and 1 mM PMSF as protease inhibitors. Homogenates were centrifuged at 15,000 x g for 10 min, and the supernatant was collected (Starting material, SM). The protein content was determined using a Bio-Rad protein assay (Bio-Rad). For coimmunoprecipitation, 1 mg of protein was brought to a volume of 500 μl with lysis buffer for immunoprecipitation (150 mM NaCl, 50 mM HEPES, and 1% Triton X-100, pH 7.4) supplemented with protease inhibitors. Samples were precleared with 50 µl of Protein A-Sepharose beads for 1 h at 4°C with gentle mixing. Next, each sample was incubated in a small chromatography column (Bio-Rad MicroBio-Spin Chromatography Columns) containing 2.5 mg of anti-GFP antibody (GeneScript) that had been previously cross-linked to Protein A-Sepharose beads for 2 h at room temperature (RT) with gentle mixing. All column centrifugation steps were performed for 30 s at 1,000 x g. Columns were washed with lysis buffer. Finally, for elution, the columns were incubated with 100 µl of 0.2 M glycine (pH 2.5) and centrifuged for 30 s at 1000 x g. SM (50 µg) and immunoprecipitates (IP) were prepared by adding 20 µl of Laemmli SDS loading buffer (5x), and the preparations were boiled and separated on 10% or 12% SDS–PAGE gels. Next, they were transferred to PVDF membranes (Immobilon-P; Millipore Sigma) and blocked with 0.05% Tween-20 PBS supplemented with 5% dry milk before immunoreaction. Filters were immunoblotted with antibodies against GFP (1:500; Roche), β-actin (1:50,000, Sigma), Na^+^/K^+^ ATPase (Developmental Studies Hybridoma Bank, The University of Iowa), VDAC (1:5.000, Calbiochem), TOMM70A (1:200, Abcam), TIMM10 (1:250, Abcam), HSP70 (1:1.000, Abcam), HSP90 (1:10,000, Abcam), TIMM50 (1:100, Abcam) or TIMM22 (1:5,000, Abcam). Finally, membranes were washed with 0.05% Tween-20 PBS and incubated with horseradish peroxidase–conjugated secondary antibodies (Bio-Rad). Irreversible cross-linking of the antibody to the Sepharose beads was performed after incubation of the antibody with Protein A-Sepharose beads for 1 h at RT. The beads were then incubated with 500 µl of 5.2 mg/ml dimethylpimelimidate (Pierce) for 30 min at RT by gentle mixing. The beads were washed with Tris-buffered saline containing 0.2 M glycine (pH 2.5) followed by Tris-buffered saline. Once these steps were performed, the columns were incubated with the protein lysates to perform the immunoprecipitation as described above. For the proteomic analysis, coimmunoprecipitation experiments were performed with Dynabeads-Protein A^®^ (Life Technologies). In this experiment, 2 mg of protein lysate were brought to a volume of 500 μl with lysis buffer supplemented with protease inhibitors. Samples were incubated for 2 h at RT or overnight at 4°C with Dynabeads^®^ cross-linked to 7.5 mg of anti-GFP antibody (GeneScript). BS3 reagent was used for antibody cross-linking according to the manufacturer’s instructions. Immunoprecipitates were washed three times and eluted with the elution buffer provided by the manufacturer. A total of 164 immunoprecipitates were independently performed and stored at 20°C. Eluates were concentrated together using methanol/chloroform precipitation, and the protein pellet was used for the HPLC–MS analysis.

### Protein Digestion, Nano-HPLC, and Mass Spectrometry (MS)

The sample was digested with trypsin using standard protocols (25). Briefly, the sample was reduced with 0.5 mM TCEP (tris(2-carboxyethyl)phosphine)/50 mM Tris, pH 7.4, for 30 min at 37°C and alkylated for 30 min with IAA in the dark. Then, digestion was performed with trypsin (0.1 μg/μl) in 25 mM Tris, pH 7.4, and 0.1% SDS at 37°C overnight. The digestion was stopped by adding formic acid. Peptides were extracted with 100% acetonitrile (ACN) and completely evaporated. Samples were reconstituted in 9 μL of 3% ACN and 1% formic acid in an aqueous solution for MS analysis. Liquid chromatography was performed using a nano-HPLC Eksigent system. HPLC separation was performed with a gradient of 3% to 35% ACN in 0.1% formic acid for 120 min. Peptides were analyzed with a Velos LCQ-Orbitrap mass spectrometer (Thermo Fisher Scientific). Data were processed using Xcalibur 2.1 (Thermo Fisher Scientific) and submitted to SEQUEST software for further analysis with the HUMAN UniProt SwissProt database.

### Bioinformatic Analysis

Cytoscape v3.7.1 software was used for the proteomic analysis of the Kv1.3 interactome (26, 27). The network of protein–protein interactions was retrieved using STRINGapp with a confidence of 95%. The Allegro weak-clustering force-directed layout was used for visualization. Functional enrichment analysis was performed using Gene Ontology, Reactome Pathways and KEGG Pathways databases. Manual annotation of interactions described in the literature was performed when indicated. For the multiple sequence alignment analysis, Clustal Omega (28) and JalView were used (29).

### Purification of Mitochondria and Flow Cytometry Analysis

Mitochondria were purified from HEK 293 cells using differential centrifugation (adapted from (30)). Briefly, 80% confluent cells were trypsinized and washed twice with PBS without Ca^2+^ and centrifuged at 600 x g for 10 min. Cells were homogenized in initial buffer 1 (225 mM mannitol, 75 mM sucrose, 0.1 mM EGTA, and 30 mM Tris, pH=7.4) and centrifuged again at 600 x g for 10 min to discard cell debris and nuclei. The supernatant was centrifuged at 7,000 x g for 10 min. The mitochondria-containing pellet was suspended in initial buffer 2 (225 mM mannitol, 75 mM sucrose, and 30 mM Tris, pH=7.4) and centrifuged again at 7,000 x g. The pellet was suspended in buffer 2 again but centrifuged at 10,000 x g to obtain the purified mitochondrial fraction. The supernatant from the first 7,000 x g centrifugation step was subsequently centrifuged at 20,000 x g for 30 min to collect a membrane-enriched pellet. Mitochondrial and membranous fractions were suspended in 50 µl of buffer 2. The supernatant was further centrifuged at 100,000 x g and concentrated with methanol/chloroform precipitation to 50 µl to obtain the cytosolic fraction. All processes were performed at 4°C. Samples were analyzed using Western blotting as described above. For flow cytometry studies, YFP-expressing mitochondrial fractions were sorted using a FACSAria FUSION instrument (BD Bioscience).

### Immunocytochemistry and Confocal Imaging

Cells seeded on poly-D-lysine-treated coverslips were used 24 h after transfection. Cells were washed with PBS and fixed with 4% paraformaldehyde (PFA) for 10 min at RT. Cells were permeabilized with 0.1% Triton X-100 for 10 min to detect HSP70 or HSP90. After a 60 min incubation in blocking solution (10% goat serum (Gibco), 5% nonfat dry milk, PBS), cells were incubated with rabbit anti-HSP70 (1:50, Abcam) or anti-HSP90 (1:200, Abcam) antibodies in 10% goat serum and 0.05% Triton X-100 for 1 h. After 3 washes, preparations were incubated for 45 min with an Alexa Fluor 660-conjugated antibody (1:200; Molecular Probes), washed and mounted using Mowiol (Calbiochem). All procedures were performed at RT. All images were acquired with a Zeiss 880 confocal microscope. The colocalization analysis was performed with ImageJ software (v 1.53m 28, National Institutes of Health, USA) as described in a previous study (31).

### Transmission Electron Microscopy (TEM)

Cells were transfected, and after 24 h, they were fixed with 4% PFA and 0.1% and glutaraldehyde at RT for 1 h followed by 2% PFA for 30 min. High-pressure freeze cryofixation with liquid N_2_ and cryosubstitution, Lowicryl resin embedding, polymerization of blocks and cutting of ultrathin sections of 60 nm were performed in collaboration with Unitat de Criomicroscòpia Electrònica (CCiTUB). Samples were mounted over Formvar-coated grills, and sections were finally stained with 2% uranyl acetate for 15 min. Immunolabeling was performed with primary anti-Kv1.3 (1:30, Neuromab), anti-HSP70 (1:50, Abcam) or anti-HSP90 (1:30, Abcam) antibodies. Secondary antibodies were conjugated to 12 and 18 nm gold particles, as indicated. Samples were imaged using a Tecnai Spirit 120 kV microscope.

### Statistics

Results are presented as the means ± SE. Student’s t test, paired t test, one-way ANOVA with Tukey’s *post-hoc* test and two-way ANOVA were used for statistical analyses (GraphPad PRISM v5.01). P < 0.05 was considered statistically significant.

## Results

### Unconventional Mitochondrial Kv1.3 Targeting

**Figure 1:**
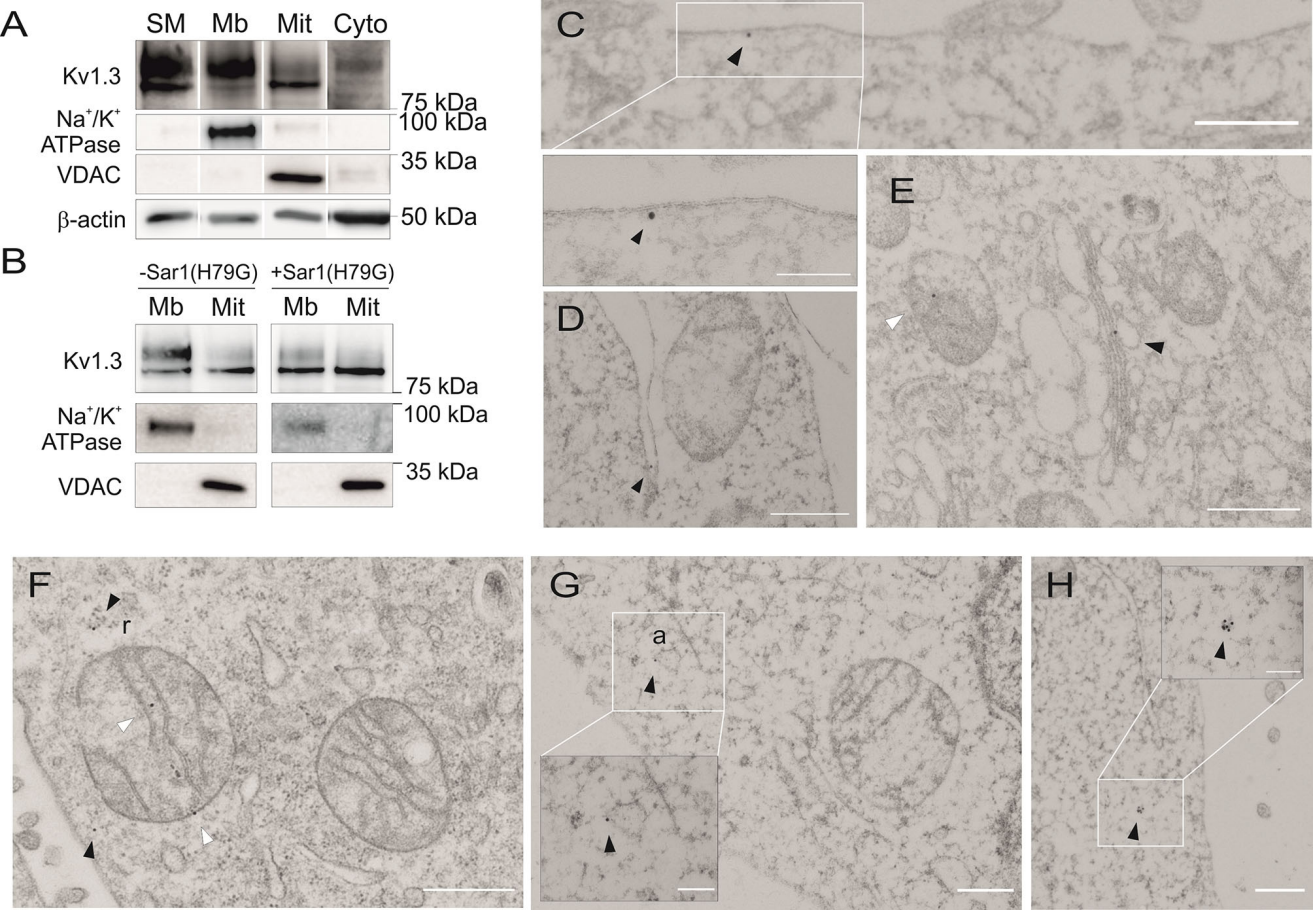
Kv1.3 is targeted to the mitochondria by bypassing the endoplasmic reticulum. **(A)** Subcellular fractionation to obtain the membranous (Mb), mitochondrial (Mit) and cytosolic (Cyto) fractions of HEK 293 cells transfected with Kv1.3YFP. SM, starting materials. Na^+^/K^+^ ATPase is used as a plasma membrane (PM) marker, and VDAC is used as a mitochondrial marker. β-actin identified cytosolic-enriched fractions. Note the difference in molecular weight of Kv1.3 bands from the Mb and Mit fractions. **(B)** Mb and Mit fractions of HEK 293 cells transfected with Kv1.3YFP were obtained. Cells were cotransfected with (+) or without (-) a constitutively active Sar1(H79G) dominant-negative GTPase. **(C–H)** Electron micrographs of HEK 293 cells transfected with Kv1.3YFP. Kv1.3 was labeled with 18 nm immunogold particles. Arrowheads indicate Kv1.3. **(C)** Plasma membrane Kv1.3. **(D)** Kv1.3 in the ER. **(E)** Kv1.3 in the Golgi (black arrowhead) and in the IMM (white arrowhead). **(F–H)** Black arrowheads indicate cytosolic Kv1.3, and white arrowheads indicate mitochondrial Kv1.3. Note the presence of Kv1.3 in ribosomes (r) close to a mitochondrion or embedded in the actin cytoskeleton (a) in **(F, G)**, respectively. The scale bar indicates 500 nm. White square insets in **(C, G, H)** highlight higher magnification regions with a scale bar representing 200 nm.

The voltage-gated potassium channel Kv1.3 is expressed both at the plasma membrane (PM) and the inner mitochondrial membrane (IMM) (10). Several forward traffic motifs regulate channel arrival at the PM (10, 23). However, the mechanisms driving mitochondrial targeting of Kv1.3 remain elusive. Kv1.3 was detected as two major forms in whole cell lysates, as shown by western blot analysis (**Figure 1A**): a fuzzy upper band, mainly corresponding to glycosylated forms, and a sharp lower and unglycosylated band (23, 32). While most plasma membrane Kv1.3 (Mb) was glycosylated, mito-Kv1.3 (Mit) was mainly unglycosylated (**Figure 1A**). The expression of Na^+^/K^+^ ATPase and VDAC (mitochondrial voltage-dependent anion channel) defined the Mb and Mit extracts, respectively. In addition, we identified some Kv1.3 proteins in the soluble cytosolic fraction (Cyto), which were enriched in β-actin.

Kv1.3 forward traffic is a COPII-dependent mechanism (23, 33). COPII vesicles are formed at ER exit sites (ERESs) and mediate ER protein export. Sar1 initiates the formation of COPII vesicles. Using a constitutively active Sar1 mutant (Sar1H79G), the forward delivery of ER proteins is prevented (23). Sar1H79G reduced the abundance of the upper mature band of PM Kv1.3 (Mb) with a minor effect on the expression of mitoKv1.3 (Mit) (**Figure 1B**). These data suggest that the mitochondrial targeting of Kv1.3 was independent of the anterograde vesicle-mediated pathway.

Electron microscopy and immunogold labeling confirmed the localization of Kv1.3 at the plasma membrane (**Figure 1C**) and in compartments associated with forward trafficking (ER, **Figure 1D**; Golgi apparatus, **Figure 1E**) and mitochondria (**Figures 1E, F**). Cytosolic forms of Kv1.3 were also identified (**Figures 1F–H**). Interestingly, Kv1.3 stained cytosolic ribosomes (r) and the actin cytoskeleton (a) near mitochondria (**Figures 1F, G**). These observations suggest that some component of Kv1.3 biogenesis is independent of conventional ER mechanisms and that the channel might be partially processed in the cytosol. Therefore, similar to other mitochondrial proteins, cytosolic Kv1.3 might bypass the ER to reach the IMM (6).

**Figure 2:**
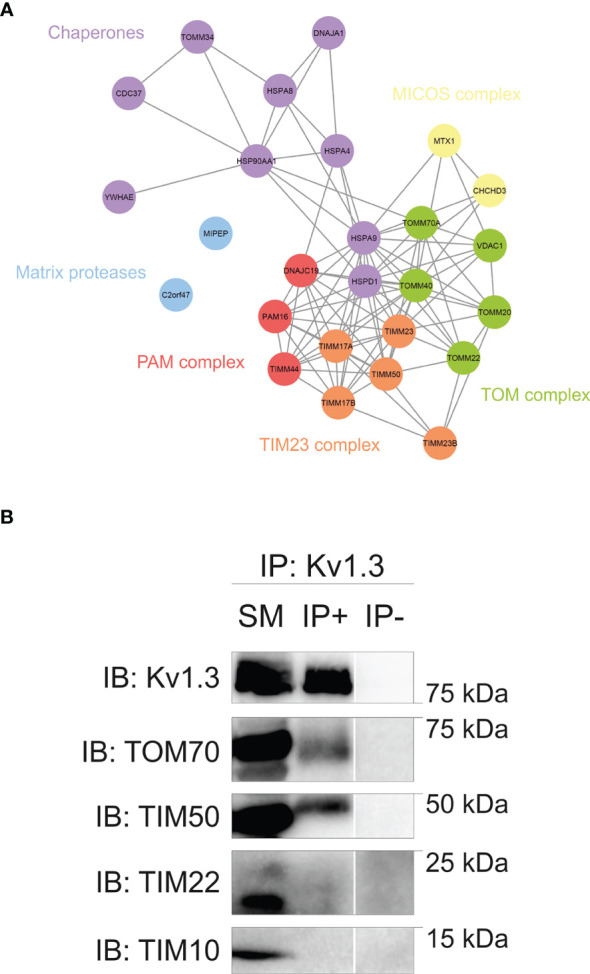
The Kv1.3 interactome reveals an unconventional mitochondrial import pathway for Kv1.3. **(A)** Network showing protein–protein interactions (PPIs) in the mitochondrial Kv1.3 interactome of HEK 293 cells transfected with Kv1.3YFP. The mitochondrial import subnetwork was obtained after a functional enrichment analysis of the Kv1.3 interactome. A force-directed layout was used for network visualization. Protein names are indicated at the node center. The color code indicates functional relations between proteins (purple, chaperones; blue, matrix proteases; red, PAM complex; orange, TIM23 complex; green, TOM complex; yellow, MICOS complex). TOMM34, CDC37 and YWHAE were manually annotated based on published evidence. **(B)** HEK 293 cells were transfected with Kv1.3YFP. Total cell lysates (SM, starting materials) were immunoprecipitated against Kv1.3 (IP+). IP-, absence of the Kv1.3 antibody. Samples were immunoblotted (IB) with antibodies against Kv1.3 and different TIM/TOM proteins, as indicated.

We used a proteomic approach to obtain additional insights into Kv1.3 mitochondrial targeting. The Kv1.3 interactome was obtained by immunoprecipitating the channel with antibody-coated Dynabeads. Coimmunoprecipitated proteins were identified using liquid chromatography–mass spectrometry (LC–MS), and the protein–protein interaction (PPI) network of the Kv1.3 interactome was analyzed. The network was retrieved using STRINGapp (https://apps.cytoscape.org/apps/stringapp) and Cytoscape (https://cytoscape.org/) software. The mitochondrial import pathway was further deciphered by performing a functional enrichment analysis. A force-directed layout was used for visualization (**Figure 2A**). Although MitoFates analysis indicated that Kv1.3 does not contain a presequence for mitochondrial targeting (34), a presequence-dependent TIM23 complex, but not presequence-independent TIM22 components, was detected. Furthermore, some cytosolic chaperones related to mitochondrial protein import were identified. These results were further confirmed using coimmunoprecipitation (**Figure 2B**). TOM70 and TIM50 – an essential member of the TIM23 complex – were associated with Kv1.3. We also confirmed the absence of an interaction between Kv1.3 and members of the TIM22 complex, such as the TIM10 receptor and the TIM22 translocase (**Figure 2B**).

### The HSP70/HSP90 Cytosolic Complex Mediates Kv1.3 Mitochondrial Import

**Figure 3:**
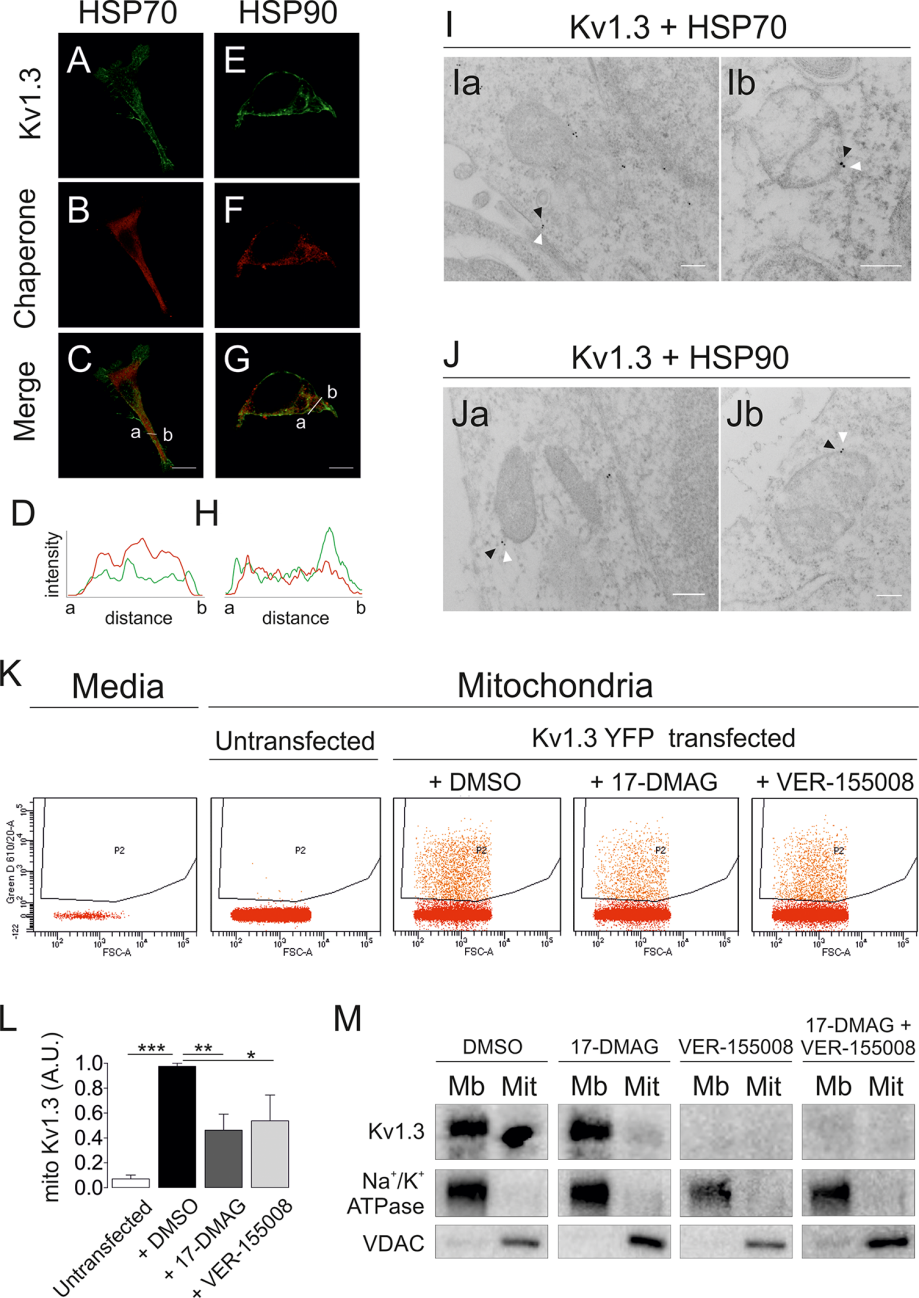
Cytosolic HSP70 and HSP90 chaperones assist with Kv1.3 mitochondrial import. HEK 293 cells were transfected with Kv1.3YFP and subsequently stained with antibodies against HSP70 and HSP90. **(A–G)** Representative confocal images showing colocalization between Kv1.3YFP (green) **(A, E)** and HSP70 **(B)** or HSP90 **(F)** (red). **(C, G)** The merged image shows colocalization in yellow. **(D, H)** Histogram showing the pixel-by-pixel analysis of the section indicated by the line in the merged Panels **(C, G)**. **(I, J)** Electron micrographs of HEK 293 cells transfected with Kv1.3YFP. Immunogold particles with sizes of 12 and 18 nm show chaperones (white arrowheads) and Kv1.3 (black arrowheads), respectively. **(I)** HSP70; **(J)** HSP90. Scale bars represent 200 nm. **(K)** HEK 293 cells transfected with Kv1.3YFP were treated with DMSO (vehicle), 20 μM VER-155008 (HSP70 inhibitor) or 1 μM 17-DMAG (HSP90 inhibitor) for 18 h Next, mitochondria were purified, and the YFP intensity was analyzed using flow cytometry. Media (DMEM) and untransfected mitochondria were used as negative controls. **(L)** Quantification of relative mitochondrial Kv1.3YFP expression upon the indicated treatments. Data are presented as the means ± SE of 3 independent experiments. *p < 0.05, **p < 0.01, and ***p < 0.001 (Student’s t test). **(M)** Immunoblot of plasma membrane (Mb) and mitochondrial (Mit) fractions of HEK 293 cells transfected with Kv1.3YFP and treated with chaperone inhibitors. Membranes were immunoblotted with antibodies against Kv1.3 (top panels), Na^+^/K^+^ ATPase (center panels) and VDAC (bottom panels).

The Kv1.3 interactome highlighted several molecular chaperones, such as HSP70 (HSPA8 and HSPA9) and HSP90, which are linked to mitochondrial import (5, 35). Kv1.3 partially colocalized with both chaperones in HEK 293 cells (**Figures 3A–H**). Electron micrographs further identified Kv1.3 in close proximity to HSP70 and HSP90. Kv1.3-HSP(70/90) dyads were located close to mitochondria (**Figures 3I, J**). We analyzed the presence of mitoKv1.3 using flow cytometry in the absence or presence of inhibitors of these chaperones to study whether HSP70 and HSP90 activities were required for Kv1.3 mitochondrial import. We isolated mitochondria from Kv1.3YFP-transfected HEK 293 cells, and the YFP signal intensity was analyzed in cells treated with 20 µM VER-155008 (HSP70 inhibitor) (36) and 1 µM 17-DMAG (HSP90 inhibitor) (37). Untransfected mitochondria and mitochondria-free media were used as negative controls (**Figure 3K**). The presence of both inhibitors decreased the abundance of mitoKv1.3 (**Figure 3L**). These results were further confirmed by detecting Kv1.3 expression in purified mitochondrial fractions (**Figure 3M**). In addition, unlike HSP90, VER-155008 treatment indicated that HSP70 also participated in the membrane targeting of Kv1.3.

### Shaker Transmembrane Domains Encode Structural Information to Mediate Mitochondrial Import

**Figure 4:**
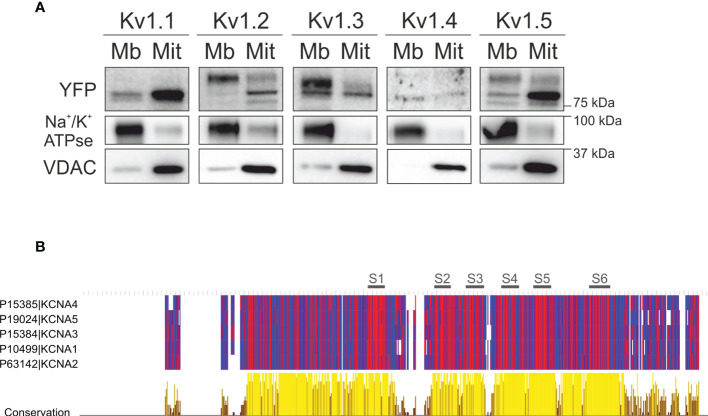
Transmembrane domains are candidate determinants of the mitochondrial import of *Shaker* channels. HEK 293 cells were transfected with Kv1.1-Kv1.5YFP channels, and mitochondrial expression was analyzed. **(A)** Membranous (Mb) and mitochondrial (Mit) fractions of HEK 293 cells transfected with the indicated member of the *Shaker* family. Note a shift in the molecular weights of the Kv1.2, Kv1.3 and Kv1.5 channels between the Mb and Mit fractions. Na^+^/K^+^ ATPse is used as a plasma membrane marker, and VDAC is used as a mitochondrial marker. **(B)** Multiple protein sequence alignment of members of the *Shaker* family of voltage-gated potassium channels performed using Clustal Omega and JalView software. UniprotKB reference numbers are indicated. Hydrophobic residues are indicated in red, and hydrophilic residues are indicated in blue. Conservation among sequences is indicated in yellow. S1-S6 black lines indicate the sequence fragments corresponding to the transmembrane segments.

**Figure 5:**
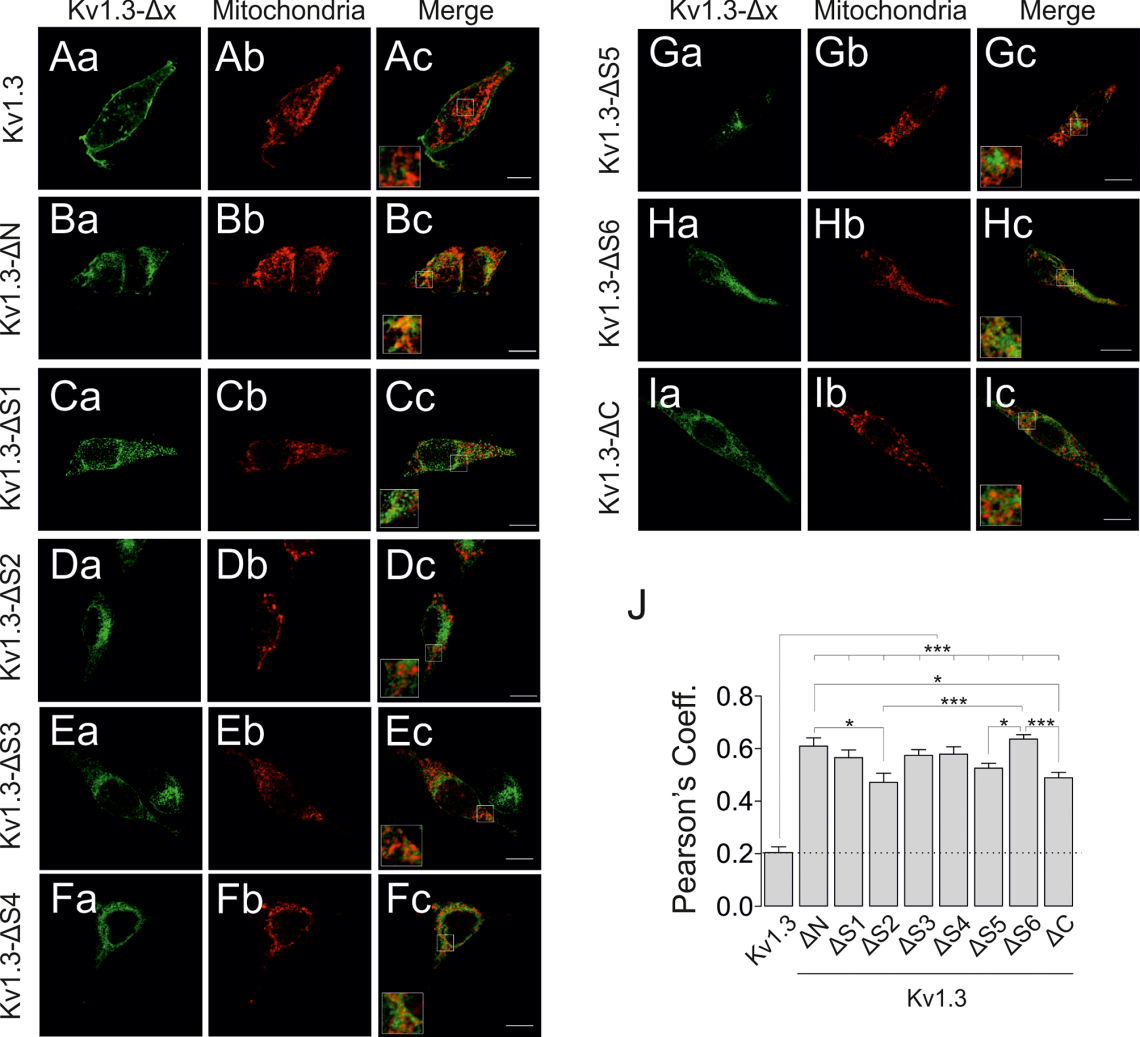
Mitochondrial targeting of Kv1.3 depends on structural information rather than on discrete motifs. HEK 293 cells were transfected with several Kv1.3-Δx channels, and mitochondrial colocalization was analyzed. **(A–I)** Representative confocal images of different Kv1.3YFP constructs (a, green) and the mitochondrial marker (b, pmitoRFP in red). Kv1.3-Δx indicates the transmembrane segment deleted from the expressed Kv1.3YFP construct. Kv1.3-ΔN **(B)** and Kv1.3-ΔC **(I)** indicate deletion of the N- and C-termini, respectively. Panel c (merge) shows colocalization in yellow. Square inset indicates the region shown at higher magnification. The scale bar represents 10 µm. **(J)** Quantification of mitochondrial colocalization using Pearson’s correlation coefficient. The dashed line highlights the Kv1.3 WT value. Data are presented as the means ± SE (n>30). *p < 0.05 and ***p < 0.001 (one-way ANOVA and Tukey’s *post hoc* test).

**Figure 6:**
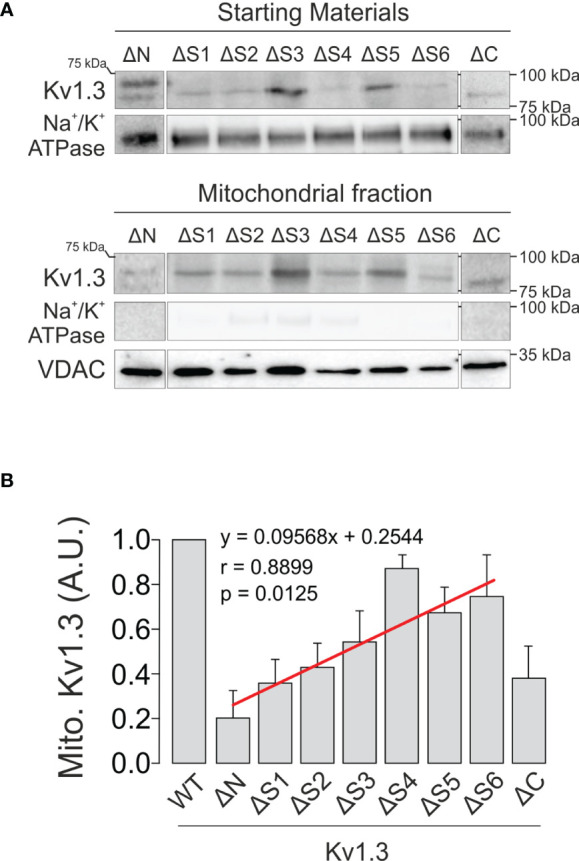
Sequential deletion of Kv1.3 segments results in differential mitochondrial targeting. HEK 293 cells were transfected with several Kv1.3-Δx channels, and mitochondrial expression was studied. **(A)** Representative immunoblot showing purified mitochondrial fractions (bottom panels) from total lysates (starting materials) of HEK 293 cells transfected with Kv1.3-Δx. Samples were immunoblotted with antibodies against YFP (Kv1.3), Na^+^/K^+^ ATPase (membrane marker) and VDAC (mitochondrial marker). **(B)** Relative mitochondrial Kv1.3-Δx expression. Data are presented as the means ± SE (n=3-5). The linear regression shown in red (y=0.09568x+0.2544) had a Pearson’s correlation coefficient of r=0.8899 (p=0.0125). Kv1.3-ΔC was not included in the calculation because of its substantial mitochondrial-independent ER retention (23).

An *in silico* analysis showed no canonical mitochondrial-targeting sequence for Kv1.3. Therefore, we aimed to further decipher the molecular determinants involved in the mitochondrial translocation of the channel. In addition to Kv1.3, Kv1.1 and Kv1.5 are targeted to mitochondria; therefore, we extended this study to other *Shaker* isoforms. Interestingly, all members (Kv1.1-Kv1.5) tested were targeted to mitochondria (**Figure 4A**). Similar to Kv1.3, the molecular weights of Kv1.2 and Kv1.5 shifted between the membrane and mitochondria, suggesting important differences in posttranslational modifications, such as glycosylation. Because the tested *Shaker* channels localized to the mitochondria, we are tempted to speculate that all isoforms might share similar targeting features. Furthermore, HSP70 and HSP90, which function in the mitochondrial import of Kv1.3, may participate in this process by interacting with the hydrophobic domains within the channel. As expected, multiple sequence alignment showed that, except for the cytoplasmic C- and N-terminal domains, protein sequences exhibited high homology among members (Kv1.1-Kv1.5) of the *Shaker* family (**Figure 4B**). An extensive hydrophobicity (red) and hydrophilicity (blue) analysis revealed a large number of conserved amino acids (yellow). The consensus was restrained to certain N-terminus portion, which contains the tetramerization domain, and the highly hydrophobic S1 to S6 transmembrane segments. In this context, we next generated a repertoire of Kv1.3 channels with selective domain deletions, including deletions in the N- and C-termini, as well as each transmembrane segment (S) (**Supplementary Figure 1**). As previously reported, confocal images showed that the C-terminus of Kv1.3 (Kv1.3 ΔC), which is essential for Kv1.3 forward trafficking (23), and the Kv1.3 ΔN, which is crucial for the tetramerization of the channel and for the interaction with caveolin (24, 38), exhibited substantial intracellular retention (**Figures 5A, B, I**). In addition, all Kv1.3 ΔS (1–6) mutants showed intracellular localization (**Figures 5C–H**), thereby indicating that alterations in any structural domain of Kv1.3 substantially affected the efficient membrane targeting of the channel. Colocalization analysis showed differential mitochondrial targeting among Kv1.3 ΔS constructs (**Figure 5J**). Pearson’s coefficients highlighted that intracellular Kv1.3 retention concomitantly correlates with an augmented mitochondrial localization of the channel, as described previously (19). We next purified VDAC-enriched mitochondrial extracts and analyzed the expression of ΔKv1.3 channels to obtain additional insights (**Figure 6A**). The channel abundance showed a steady dependence on the proximal segments of the channel. Thus, as the first domain structures of the channel are preserved, mitochondrial expression is increased (**Figure 6B**). Our data suggested that although no specific domain was essential for mitochondrial targeting, collectively, all domains were sequentially involved in IMM translocation of the channel.

**Figure 7:**
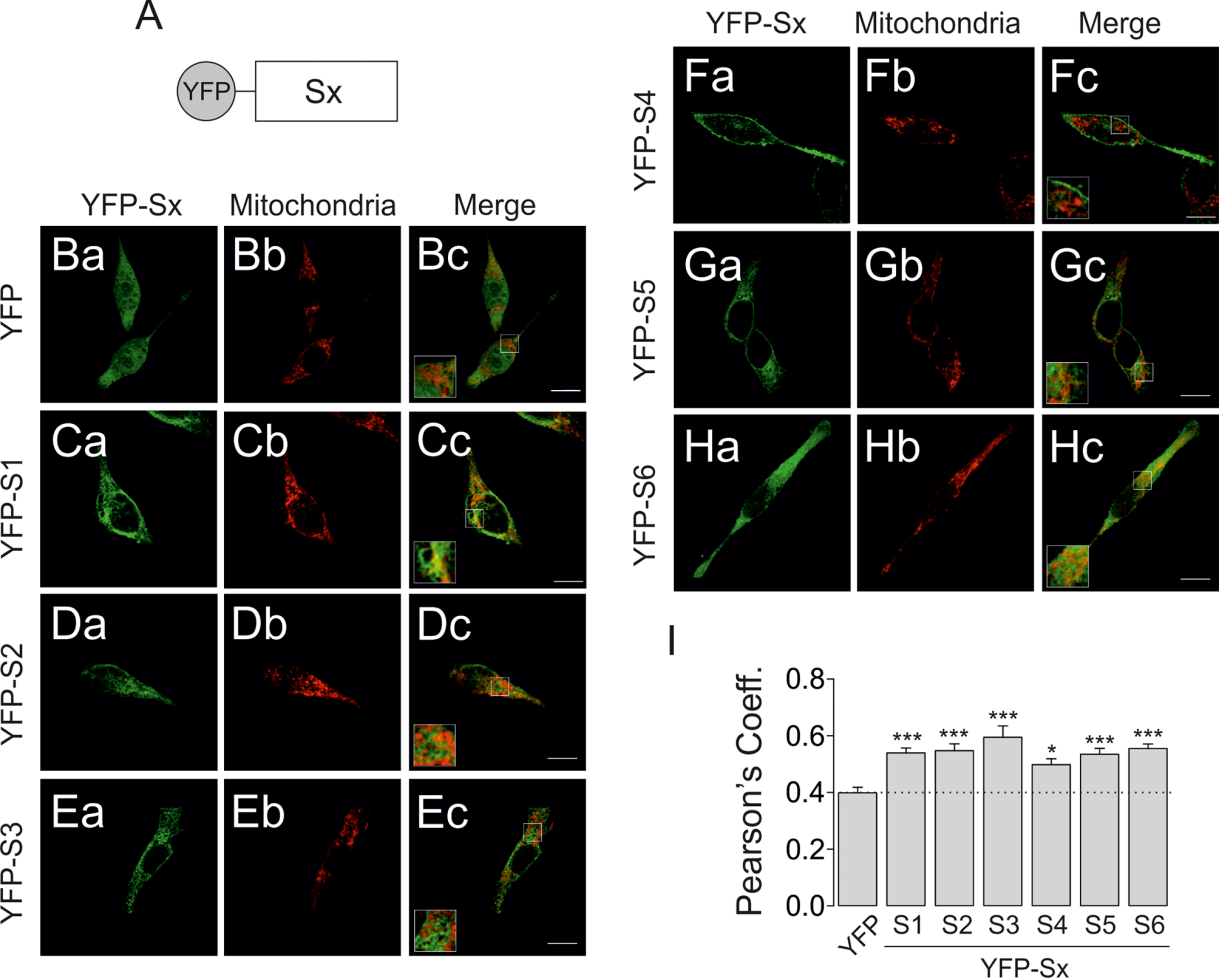
Transmembrane segments encode sufficient information to localize Kv1.3 to mitochondria. HEK 293 cells were transfected with several YFP-Sx(1-6) transmembrane peptides, and mitochondrial colocalization was analyzed. **(A)** Cartoon representing the transmembrane peptide constructs. Sx indicates the transmembrane segment (1-6) inserted in the YFP plasmid. **(B–H)** Representative confocal images of HEK 293 cells cotransfected with different YFP constructs (a, green) and a mitochondrial marker (b, pmitoRFP in red). The merged image (c) shows colocalization in yellow. The square inset indicates the region shown at higher magnification. The scale bar represents 10 µm. **(I)** Quantification of colocalization using Pearson’s correlation coefficient. Data are presented as the means ± SE (n>30). *p < 0.05 and ***p < 0.001 (one-way ANOVA compared with YFP).

**Figure 8:**
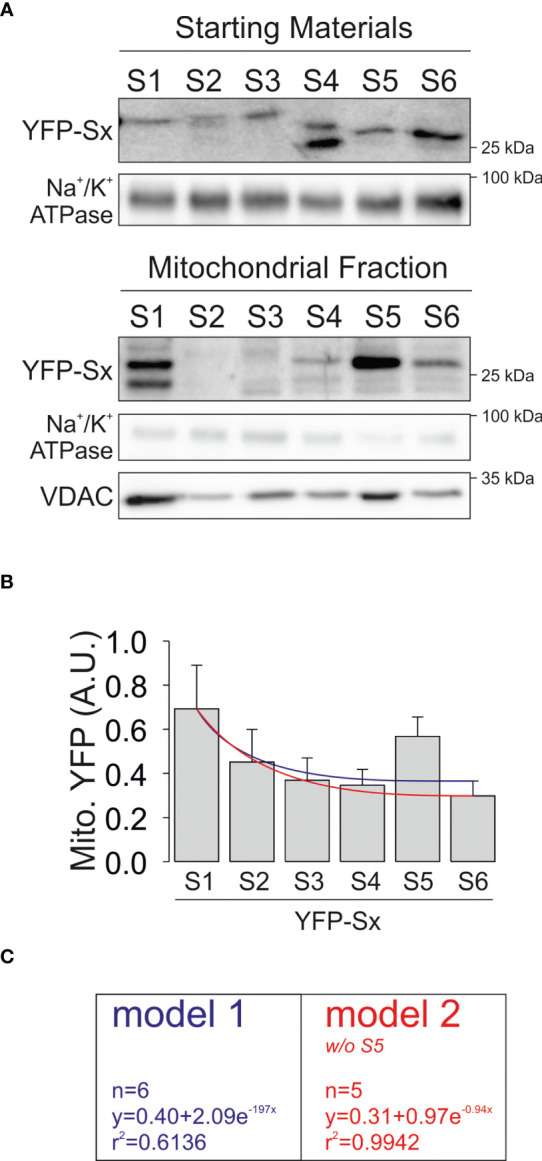
Mitochondrial expression of YFP-Sx(1-6) transmembrane peptides. HEK 293 cells were transfected with several YFP-Sx(1-6) transmembrane peptides, and mitochondrial expression was studied. **(A)** Representative immunoblots showing purified mitochondrial fractions from total lysates (starting materials) and mitochondrial extracts (mitochondrial fractions). Samples were immunoblotted with antibodies against YFP (YFP-Sx(1-6)), Na^+^/K^+^ ATPase (membrane marker) and VDAC (mitochondrial marker). **(B)** Relative mitochondrial YFP-Sx expression is presented in arbitrary units (A.U.). Data are presented as the means ± SE (n=3-5). Blue line, nonlinear regression curve representing a monoexponential decay with all constructs. Red line, nonlinear regression curve representing a monoexponential decay without S5. Note that with slight variations, both lines represent a bona fide fitting of data. **(C)** Calculations of both fits. Model 1 (blue) includes values from all YFP-Sx and Model 2 (red) lacks S5.

Based on this information, we next generated a series of YFP-chimeric constructs individually containing the S1 through S6 transmembrane domains of Kv1.3 and analyzed their mitochondrial targeting (**Figure 7A**). Confocal images showed that all constructs exhibited increased mitochondrial localization compared to YFP alone (**Figures 7B–H**). Therefore, all YFP-Sx individually contained information driving the channel to the mitochondria (**Figure 7I**). Finally, we analyzed the levels of all Kv1.3-Sx proteins in VDAC-enriched mitochondrial extracts (**Figure 8A**). The protein abundance of the Kv1.3-Sx constructs consistently indicated lower mitochondrial expression of constructs more distal from the N-terminus of the channel (**Figures 8B, C**). The intriguing behavior of Kv1.3-S5 prompted us to calculate two alternative possibilities. However, both models yielded similar monoexponential decays with better bona fide fitting in Model 2 (r^2 =^ 0.9942).

## Discussion

The voltage-gated Kv1.3 channel exhibits dual localization both at the plasma membrane and the mitochondria. The balance between the two destinies determines cell survival and might be especially critical for cancer cells. Thus, deciphering the mechanisms involved in the spatial localization of the channel is of physiological and pathological relevance. While the molecular determinants of cell surface targeting are constantly being investigated, no study has addressed Kv1.3 mitochondrial translocation. In the present study, we described, for the first time, the mechanisms driving Kv1.3 translocation to mitochondria. Strikingly, Kv1.3 uses an unconventional presequence-independent pathway to reach the inner mitochondrial membrane. We identified that the transmembrane domains function as redundant motifs mediating the import process. The interaction with cytosolic HSP70/HSP90 chaperones is essential prior to an association with the unconventional TOM/TIM23 mitochondrial translocation machinery.

**Figure 9:**
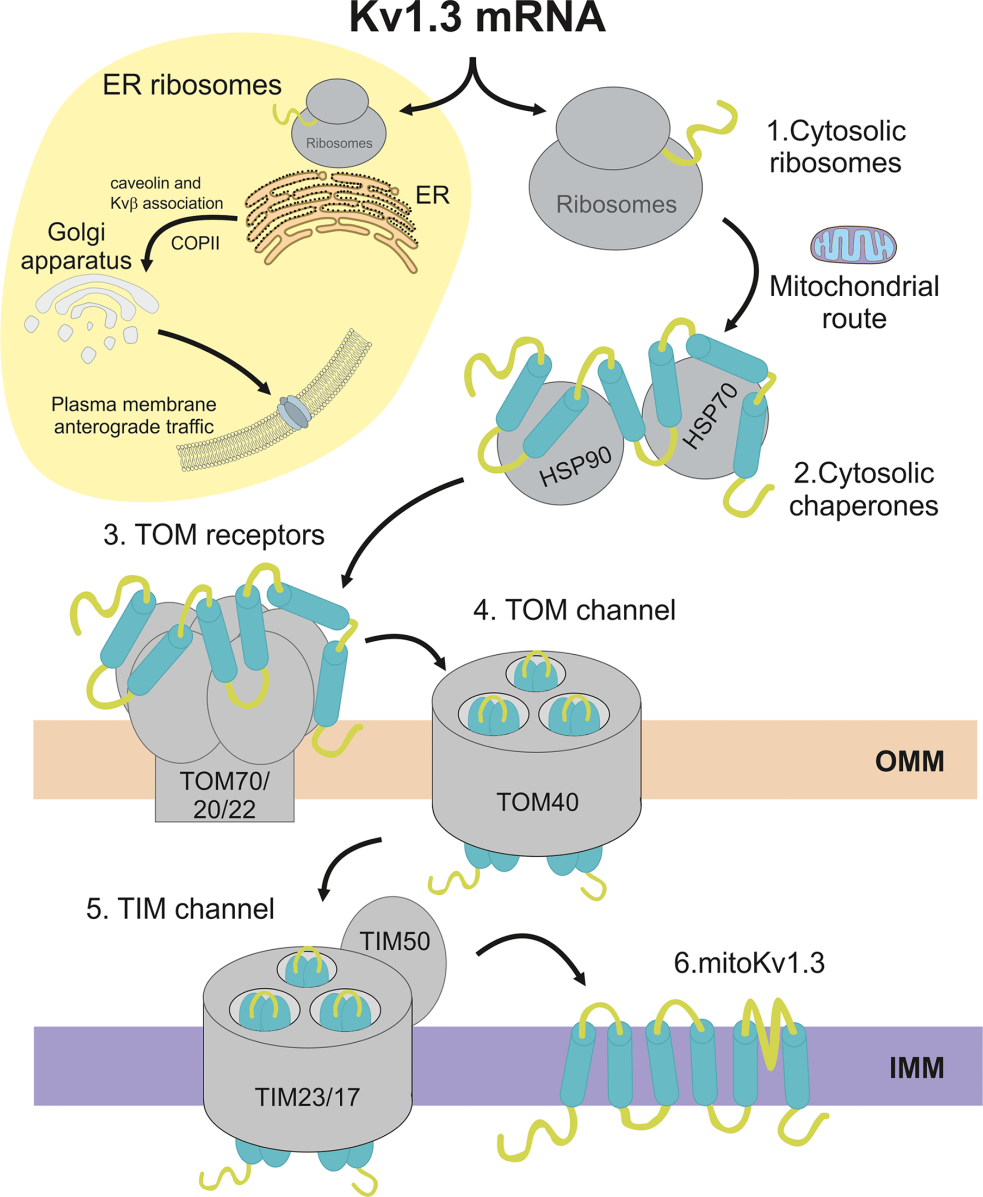
Schematic representation of the mitochondrial import pathway for Kv1.3. The Kv1.3 channel is cotranslated by ER-linked and cytosolic ribosomes. ER ribosomes synthesize the plasma membrane Kv1.3, which interacts early with ancillary proteins, such as Kvβ subunits and caveolin, and is routed to the cell surface *via* COPII-dependent anterograde trafficking. The yellow circle restrains anterograde trafficking to the plasma membrane. Alternatively, **(1)** the Kv1.3 mRNA, which is translated by cytosolic ribosomes, follows the mitochondrial route. **(2)** By interacting with channel hydrophobic domains, cytosolic chaperones and cochaperones provide Kv1.3 with a partially folded state competent for mitochondrial translocation. Sequential transmembrane domains from the N- to C-terminus cooperate to achieve the proper folded state. **(3)** TOM receptors recognize cytosolic Kv1.3. **(4)** The TOM40 channel at the OMM translocates Kv1.3 to the TIM complex at the IMM. Finally, **(5)** the TIM50 receptor facilitates Kv1.3 translocation through the TIM23/17 channel into the IMM **(6)**. Spatial and temporal mechanisms defining Kv1.3 membrane topology and tetramerization at the IMM remain unknown.

Kv1.3 is translationally translocated into the ER for forward traffic to the cell surface (23, 39). Mitochondria and ER are intimately connected, but unlike lipids, protein transfer from the ER to mitochondria has not been reported. However, glycosylated proteins have already been detected in chloroplasts (40). Our results indicate that, similar to the viral Kesv channel (20), Kv1.3 translocated to the mitochondrion directly from the cytosolic side, thus bypassing the ER (**Figure 9**). Similarly, we identified cytosolic membrane-free Kv1.3 units that are usually embedded within ribosome pools and the actin filament network, which raised some questions. Does Kv1.3 approach the OMM co or posttranslationally? Evidence indicates that some proteins are imported into mitochondria after complete synthesis in cytosolic ribosomes. However, some documentation revealed clusters of ribosomes at the OMM using electron cryo-tomography (4, 41). Protein translocation requires a partially unfolded state because TOM and TIM translocases form narrow channels that do not accommodate bulky conformations (8). Chaperones would facilitate this process (**Figure 9**). The Kv1.3 interactome contained several cytosolic chaperone and cochaperone proteins that potentially participate in Kv1.3 mitochondrial translocation. HSP70 and HSP90 emerged as essential mediators of this process. Unfolded proteins expose several hydrophobic binding sites for HSP70, while HSP90 interacts with the surface of partially folded proteins (35, 42). Both chaperones cooperate to deliver cargo proteins to TOM complex receptors (5). Functional enrichment analysis of the Kv1.3 interactome revealed other chaperones and cochaperones relevant for mitochondrial import, such as Tomm34 and Cdc37, both cochaperones of the HSP70/HSP90 complex. Tomm34 requires a fully translated peptide to associate with the chaperone complex, which would indicate posttranslational mitochondrial import of Kv1.3 (43, 44). Mitochondrial import stimulation factor (MSF) was also detected (YWHAE). MSF is a 14-3-3ε adaptor protein that functions as a cytoplasmic chaperone to target precursor proteins to mitochondria (45).

In addition to cytosolic chaperones, components of the TOM complex were also identified, such as Tom40, Tom70, Tom20 and Tom22 (**Figure 9**). The latter three proteins are the receptor components of the TOM complex, while Tom40 constitutes the so-called general insertion pore that drives the OMM insertion of β-barrel proteins such as VDAC (46, 47). Tom70 recognizes internal hydrophobic targeting sequences, and Tom20/Tom22 are receptors for proteins with cleavable N-terminal targeting sequences. Interestingly, the main components of the TIM23 complex, including Tim23, Tim17 and the receptor subunit Tim50, were also identified. These results suggest an unconventional pathway for mitochondrial Kv1.3 translocation. Kv1.3 is a multispanning protein of the IMM that does not contain a cleavable N-terminal presequence. Thus, its import through the TIM22 complex was expected. In this scenario, the Kv1.3 mechanism might be broader than expected because the Kesv channel and Sym1 protein, which share a multispanning α-helix architecture with Kv1.3, are also routed into the IMM through a presequence-independent TIM23 mechanism (20, 48).

We developed two molecular strategies to decipher the determinants involved in mitochondrial targeting of channels. *Shaker* channels, all of which expressed in mitochondria, exhibit highly hydrophobic and conserved transmembrane segments. Therefore, these domains became good candidates because cytosolic chaperones interact with hydrophobic domains. Successive deletion of all individual downstream domains yielded similar levels of Kv1.3 mitochondrial colocalization. However, protein expression indicated that the strongest translocation signals were located at the beginning of the protein. Complementarily, individual transmembrane domains efficiently targeted to mitochondria, further indicating that redundant signals exist throughout the transmembrane domains of Kv1.3. Our results indicate that continuous proximal to distal protein synthesis accumulates interacting signals defining the mitoKv1.3 fate. Evidence has indicated that Kv1.3 transmembrane segments cooperate to mediate membrane translocation and integration of the channel and, overall, define the channel architecture in mitochondria (49). Little is known about the initial steps of potassium channel biogenesis, but the second transmembrane domain (S2) likely functions as the initial signal sequence for Kv1.3 ER translocation. S2 allows the nascent channel to remain longer in the cytosol before being translocated into the ER (49). In addition, the association with ancillary proteins, such as Kvβ regulatory subunits or caveolin, at the early N-terminus of Kv1.3 determines the plasma membrane anterograde trafficking of the channel (**Figure 9**). Our results are consistent with those findings because they suggest that mitochondrial targeting may be promoted even in the absence of any (mito) domain in the Kv1.3 sequence. Thus, translocation into mitochondria is a continuum of processes during Kv1.3 translation. Even when large amounts of Kv1.3 are synthesized, more mitochondrial-associated chaperone interacting signals are available, which would ensure efficient targeting to the final destination. These observations suggest a cotranslational mechanism of Kv1.3 alternating conventional anterograde trafficking with mitochondrial translocation. Our results extend to related Kv isoforms, either *Shaker*, such as Kv1.5 and Kv1.1, or *KCNQ* channels, such as Kv7.4, which share homology within hydrophobic transmembrane domains and target to mitochondria (11, 17). Additionally, the fourth transmembrane segment, when individually fused to YFP, nicely decorates the plasma membrane. This result, although striking, is supported by the fact that S4 strongly interacts with phospholipid membranes and partially integrates into the membrane when expressed independently (49, 50).

In conclusion, our work describes for the first time the Kv1.3 mitochondrial translocation pathway (**Figure 9**). We propose a mechanism involving multiple signals widely dispersed throughout the channel sequence that regulate the equilibrium between PM and mitochondrial Kv1.3. The expression of a full Kv1.3 protein at the ER promotes forward trafficking to the cell surface through a mechanism promoted by some ancillary interactions. A tempting speculation is that cytosolic chaperones might mask anterograde traffic determinants throughout the channel, thereby facilitating mitochondrial targeting. Channel domains cooperate and likely interact with each other to control the mitochondrial routing of Kv1.3. Further studies are needed to decipher the final channel architecture once in the IMM and the turnover mechanisms of the mitoKv1.3 channel. Our work provides insights into the mechanisms regulating the plasma membrane/mitochondrial equilibrium, which balances the final Kv1.3 destination and is essential for cell survival and apoptosis.

## Data Availability Statement

The original contributions presented in the study are included in the article/**Supplementary Material**. Further inquiries can be directed to the corresponding author.

## Ethics Statement

The studies involving human participants were reviewed and approved by The Ethics Committee of the Universitat de Barcelona and the Banc de Sang i Teixits de Catalunya approved protocols (IRB00003099). The patients/participants provided their written informed consent to participate in this study.

## Author Contributions

JC, AM, and MN-P performed experiments. JC and AF interpreted and supervised data. JC and MN-P analyzed and interpreted data. JC, IS, and AF designed, interpreted and wrote the paper. All authors discussed the work. All authors contributed to the article and approved the submitted version.

## Funding

Supported by the Ministerio de Ciencia e Innovación (MICINN/AEI), Spain (BFU2017-87104-R, PID2020-112647RB-I00 and 10.13039/501100011033) and the European Regional Development Fund. JC and MN-P hold fellowships from the Fundación Tatiana Pérez de Guzmán el Bueno and MICINN, respectively.

## Conflict of Interest

The authors declare that the research was conducted in the absence of any commercial or financial relationships that could be construed as a potential conflict of interest.

## Publisher’s Note

All claims expressed in this article are solely those of the authors and do not necessarily represent those of their affiliated organizations, or those of the publisher, the editors and the reviewers. Any product that may be evaluated in this article, or claim that may be made by its manufacturer, is not guaranteed or endorsed by the publisher.
