# Supplementary material for: The Mitochondrial Routing of the Kv1.3 Channel
Source: Front Oncol. 2022 Mar 24;12:865686. doi: 10.3389/fonc.2022.865686 (PMC8990977; doi:10.3389/fonc.2022.865686)
Supplement: Supplementary file 1 [file DataSheet_1.pdf]

Supplementary Material

The mitochondrial routing of the Kv1.3 channel

Jesusa Capera, María Navarro-Pérez, Anne Stine Moen, Ildiko Szabó and Antonio Felipe*

***Corresponding author:** Antonio Felipe, Dpt. de Bioquímica i Biomedicina Molecular, Institut de Biomedicina (IBUB), Universitat de Barcelona, Avda. Diagonal 643, E-08028 Barcelona, Spain. Tel: 34 934034616; fax: 34 934021559. E-mail address: afelipe@ub.edu

This file contains:

Supplementary Figure 1

Supplementary Table 1 and 2

Supplementary Figure.

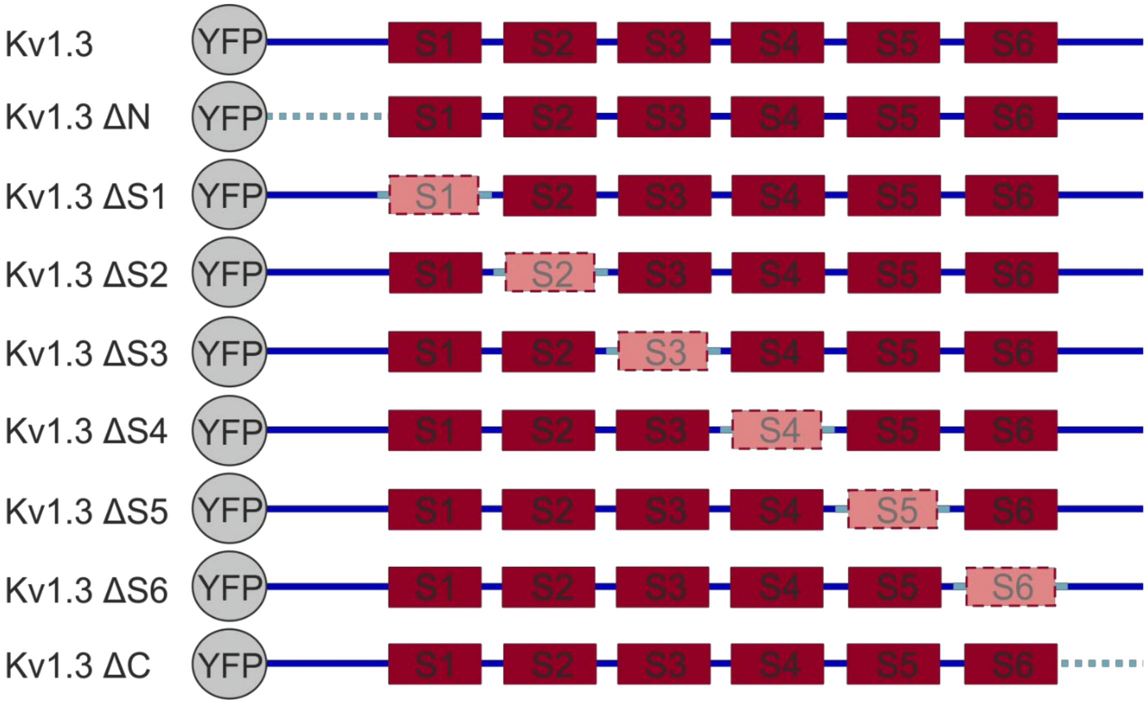

Supplementary Figure 1. Representative cartoon showing the Kv1.3ΔS(1-6) channels generated. Each of the transmembrane segments (S1-6) was individually deleted from the Kv1.3 sequence and subcloned into the pEYFP-C1 plasmid. In addition, the N- and C-termini were individually deleted, as described previously. Dotted patterns indicate deleted segments.

Supplementary Tables.

**Primer**	**Sequence (5' --> 3')**
F_rKv1.3NtXhoIS1	CCGAGAGCTCCGGGCCGGCtCGaGGCATTGCCATCGTGTCAGTGC
F_rKv1.3S1XhoIL1	TCATCTTCTGCTTGGAGACtCgAgCCGAGTTTCGCGACGAGAAGGAC
F_rKv1.3L1XhoIS2	GGGCCTCCTCTcGAGCCTCCAGCTTCTCGGACCC
F_rKv1.3S2XhoIL2	GCTGCTGGTGCGATTCTTTGCTcGagCCAGTAAAGCCACCTTCTCC
F_rKv1.3L2XhoIS3	GCCCCAGTAAAGCCACCTTCTCtcGAgATATCATGAACCTGATAGAC
F_rKv1.3S3XhoIL3	TCTGGGCACTGAGCTGGCTGctCGAgAGGGTAATGGGCAGCAGGC
F_rKv1.3L3XhoIS4	AATGGGCAGCAGGCTATGTCtCgaGCCATCCTGAGGGTCATCCGC
F_rKv1.3S4XhoIL4	AGCTCTCCCGCCATTCTcgaGGGCTGCAGATCCTGGGACAGACAC
F_rKv1.3L4XhoIS5	CAGACACTGAAGGCTTCCActCGAGAGCTGGGGCTGCTCATTTTC
F_rKv1.3S5XhoIL5	TCTCCAGTGCAGTCTACTTTGCTcgaGCAGACGACCCTTCTTCGG
F_rKv1.3L5XhoIS6	TGGTGATATGCACCCAGTGACCActcGAGGCAAGATTGTGGGCTCTC
F_rKv1.3S6XhoICt	AATTACTTCTACCctCGaGAGACAGAAGGGGAAGAGCAAGCCCAG

Supplementary Table 1. Primers used for the generation of the Kv1.3-ΔSx constructs. Kv1.3-ΔSx constructs were generated by inserting XhoI sites at the beginning and at the end of the Sx sequence in the Kv1.3 pEYFP-C1 plasmid.

Name	Sequence (5' --> 3')
F_S1	**AATTC**TGGCATTGCCATCGTGTCAGTGCTGGTCATTCTCATCTCCATTGTCATCTTCTGCTTGGAGACACTACCCTAAG
R_S1	**GATCC**TTAGGGTAGTGTCTCCAAGCAGAAGATGACAATGGAGATGAGAATGACCAGCACTGACACGATGGCAATGCCAG
F_S2	**AATTC**TGCCTCCAGCTTCTCGGACCCCTTCTTCGTAGTGGAGACCCTGTGCATCATCTGGTTCTCCTTTGAGCTGCTGGTGCGATTCTTTGCTTGCCCCTAAG
R_S2	**GATCC**TTAGGGGCAAGCAAAGAATCGCACCAGCAGCTCAAAGGAGAACCAGATGATGCACAGGGTCTCCACTACGAAGAAGGGGTCCGAGAAGCTGGAGGCAG
F_S3	**AATTC**TAATATCATGAACCTGATAGACATTGTAGCCATCATCCCTTATTTTATTACTCTGGGCACTGAGCTGGCTGAGCGACAGTAAG
R_S3	**GATCC**TTACTGTCGCTCAGCCAGCTCAGTGCCCAGAGTAATAAAATAAGGGATGATGGCTACAATGTCTATCAGGTTCATGATATTAG
F_S4	**AATTC**TCTGGCCATCCTGAGGGTCATCCGCCTAGTAAGGGTCTTCCGCATCTTCAAGCTCTCCCGCCATTCTAAGTAAG
R_S4	**GATCC**TTACTTAGAATGGCGGGAGAGCTTGAAGATGCGGAAGACCCTTACTAGGCGGATGACCCTCAGGATGGCCAGAG
F_S5	**AATTC**TGAGCTGGGGCTGCTCATTTTCTTCCTTTTCATTGGGGTCATCCTTTTCTCCAGTGCAGTCTACTTTGCTGAGTAAG
R_S5	**GATCC**TTACTCAGCAAAGTAGACTGCACTGGAGAAAAGGATGACCCCAATGAAAAGGAAGAAAATGAGCAGCCCCAGCTCAG
F_S6	**AATTC**TATAGGAGGCAAGATTGTGGGCTCTCTTTGTGCCATCGCAGGTGTCTTGACCATTGCATTGCCGGTTCCTGTGATTGTTTCCAACTTCAATTACTTCTACCACTAAG
R_S6	**GATCC**TTAGTGGTAGAAGTAATTGAAGTTGGAAACAATCACAGGAACCGGCAATGCAATGGTCAAGACACCTGCGATGGCACAAAGAGAGCCCACAATCTTGCCTCCTATAG

Supplementary Table 2. Primers used for the generation of the YFP-Sx constructs. YFP-Sx constructs were obtained by fusing the Sx segment to the C-terminus of pEYFP-C1 (Clontech). Forward and reverse oligonucleotide strands containing the Sx sequence were designed with EcoRI and BamHI sites at the beginning and at the end, respectively, to generate Sx segments.
